# Pure Food

**Published:** 1910-04-02

**Authors:** 


### The Hospital

A Journal of

The Medical Sciences and Hospital Administration.

New Series. No. 163, Vol. VII. [No. 1235. Vol. XLVIII.] SATURDAY, APRIL 2, 1910.

### Pure Food.

In these days, when Socialism is so much discussed and so little understood, the doctrine that the State should interfere in matters that concern the individual as a unit of the community is liable to be interpreted in different ways. There can, however, be little question that all interpreters, no matter whether they take up the strict Socialistic attitude, or merely admit the safeguarding clause that interference is justified only when individual liberty is likely to endanger the commonalty, must agree that in matters affecting the general health of the community, State intermeddling is necessary and indispensable. No community can permit a private owner to risk the lives of his neighbours by permitting his backyard to be used as a camping ground for patients afflicted with small-pox, or by polluting the atmosphere with an open cesspool. Similarly, no community can allow a tradesman to risk the health of his customers by selling adulterated foodstuffs, the consumption of which is fraught with danger. The small-pox sufferers must be promptly isolated, the cesspool filled up, and the condemned foodstuffs destroyed. On these points we are all agreed, and a medical officer of health who would venture to dally in taking the necessary steps to deal with such nuisances would speedily lose his billet. But when it comes to minor matters we are not so united in agreement. The proof of this is the fact that the State in this country has as yet taken no steps to safeguard the public from quackery, and that food adulteration on a large scale is still a flourishing business in these islands. It is an old but very true adage that says one half of the world knows not how the other half lives. A prominent member of the Cabinet is apparently unaware that horse-flesh is an article of human consumption in this country, and, doubtless, there are many public men who have no notion to what extent food adulteration prevails. We have no case whatever against the consumption of horse-flesh, which, so long as it is good, sound horse-flesh, is eminently suitable for food; but the case against adulteration is a strong one, and no opportunity should be lost of showing the public how strong it really is.

Other countries have dealt summarily with the matter, even to the extent of entering into competition with private traders. If an argument were needed for the desirability of municipal trading the supporters of this fine theory cannot do better than turn to the Budapest bakeries. In that city the bakers adulterated their dough with 200 per cent. of water and potato paste. They sold loaves of the much maligned black bread at a price of 50 heller per kilogram, when the intrinsic value of their wares, as foodstuffs, was perhaps a third of this price. The municipality stepped in, established model bakeries, where black bread was made from pure, unadulterated flour, and undersold the bakers. The result has been eminently satisfactory. The tradesmen lowered their prices, but found that the public still preferred to buy municipal bread which was guaranteed pure, and in the end the model bakeries have practically killed flour adulteration in Budapest. At Vienna municipal interference, on somewhat similar, though not identical lines, has stamped out milk adulteration—a consummation which London, notwithstanding the watchfulness of inspectors and the frequency of police court proceedings against hardened offenders, has not yet been able to achieve.

In these circumstances it is gratifying to note the efforts which private enterprise is making to deal with the problem over here. Such efforts can, of course, only be educative. It is only by educating the public to a sense of the importance of dealing with the matter that legislative action can be finally obtained to eradicate impure foods. It is eminently satisfactory to find that the Pure Food Exhibition, which is to be held in May, has obtained promising support from all quarters. Such an exhibition should do much to stimulate a wholesome interest in the matter of preventive action against adulteration. By showing that the unadulterated article is as economical as the adulterated, and that the advantages of the former far surpass those of the latter, an appeal will at once be made to the common-sense of every visitor to the exhibition. One regret, perhaps, is, as Mr. Sims points out, that there is no opportunity of showing the direct evils resulting from the consumption of impure food. These results are not so striking and dramatically imposing as some readers may think.

One can go on using sanded sugar and carrot pulp "strawberry jam" without becoming a confirmed dyspeptic, and too much stress must not, therefore, be laid upon the immediate perils of adulterated foodstuffs, especially as nowadays the "blender" is careful not to mix his honest nucleus with directly harmful ingredients; though it is true one or two striking instances might be cited to the contrary. The danger to the community from the consumption of such impure foods, however, is a real one in so far as it means underfeeding, insufficient feeding, and expensive feeding. These, in the long run, must inevitably produce physical deterioration, and it is a nice point how far the decay of stamina which is so frequently alluded to is the result of adulteration of foodstuffs. The economic factor in this, as in many social and some hygienic questions, is important, and it is just by demonstrating the fallacy that good food is expensive food and cheap food necessarily bad, that exhibitions such as these will do excellent service. After all, the chief weapon against the adulterator lies in the public demand for wholesome food. Once the consumer learns that he can obtain what he wants he will no longer consent to be put off with inferior stuff. If such inferior food is still foisted upon him, then there is some chance that an indignant community will clamour for legislative action—and get it.

